# The Arab world’s contribution to solid waste literature: a bibliometric analysis

**DOI:** 10.1186/s12995-015-0078-1

**Published:** 2015-09-17

**Authors:** Sa’ed H. Zyoud, Samah W. Al-Jabi, Waleed M. Sweileh, Suleiman Al-Khalil, Shaher H. Zyoud, Ansam F. Sawalha, Rahmat Awang

**Affiliations:** Poison Control and Drug Information Center (PCDIC), College of Medicine and Health Sciences, An-Najah National University, Nablus, 44839 Palestine; Department of Clinical and Community Pharmacy, College of Medicine and Health Sciences, An-Najah National University, Nablus, 44839 Palestine; WHO Collaborating Centre for Drug Information, National Poison Centre, Universiti Sains Malaysia (USM), Penang, 11800 Malaysia; Department of Pharmacology and Toxicology, College of Medicine and Health Sciences, An-Najah National University, Nablus, 44839 Palestine; Department of Medical Laboratory Sciences, College of Medicine and Health Sciences, An-Najah National University, Nablus, 44839 Palestine; Civil Engineering Department, Graz University of Technology, Graz, Austria

**Keywords:** Solid waste, Bibliometric, SCI, Arab world

## Abstract

**Background:**

Environmental and health-related effects of solid waste material are considered worldwide problems. The aim of this study was to assess the volume and impact of Arab scientific output published in journals indexed in the Science Citation Index (SCI) on solid waste.

**Methods:**

We included all the documents within the SCI whose topic was solid waste from all previous years up to 31 December 2012. In this bibliometric analysis we sought to evaluate research that originated from Arab countries in the field of solid waste, as well as its relative growth rate, collaborative measures, productivity at the institutional level, and the most prolific journals.

**Results:**

A total of 382 (2.35 % of the overall global research output in the field of solid waste) documents were retrieved from the Arab countries. The annual number of documents published in the past three decades (1982–2012) indicated that research productivity demonstrated a noticeable rise during the last decade. The highest number of articles associated with solid waste was that of Egypt (22.8 %), followed by Tunisia (19.6), and Jordan (13.4 %). the total number of citations over the analysed years at the date of data collection was 4,097, with an average of 10.7 citations per document. The *h*-index of the citing articles was 31. Environmental science was the most researched topic, represented by 175 (45.8 %) articles. *Waste Management* was the top active journal. The study recognized 139 (36.4 %) documents from collaborations with 25 non-Arab countries. Arab authors mainly collaborated with countries in Europe (22.5 %), especially France, followed by countries in the Americas (9.4 %), especially the USA. The most productive institution was the American University of Beirut, Lebanon, with 6.3 % of total publications.

**Conclusions:**

Despite the expected increase in solid waste production from Arab world, research activity about solid waste is still low. Governments must invest more in solid waste research to avoid future unexpected problems. Finally, since solid waste is a multidisciplinary science, research teams in engineering, health, toxicology, environment, geology and others must be formulated to produce research in solid waste from different scientific aspects.

## Background

Environmental and health-related effects of solid waste material are considered worldwide problems [1–3]. The exponentially increasing population, industrialization and urbanization created an increasing challenge on the management of solid waste materials for most governments. Municipal solid waste, food waste, sludge, electrical waste, construction and demolition waste are considered the main and most important waste materials that modern societies need to manage and dispose [1, 4–8]. It is important to mention that there is an enormous gap between developing countries and developed countries in disposal and management of solid waste materials.

Currently, many developing countries are devoting more efforts to improve their solid waste management. Furthermore, more scientific research activity has been observed in these countries in this regard [1, 4–8]. The Arab region generates nearly 250,000 tons of solid waste material per day. The amount of solid waste materials generated differs from one Arab country to another, and also among the regions within the same country. The generation of municipal solid waste per capita in some Arab cities, such as Kuwait, and Abu Dhabi, is over 1.5 kg per day [9, 10]. The quantities of solid waste materials generated are correlated with the rate of increase in population, economic, industrial and urban development. The predicted figure of the amounts of municipal solid waste in Arab region in 2020 will overreach 200 million tons per year [11]. Over the last years, several researchers have tried to evaluate the outcome of scientific output from Arab and non-Arab countries [12–17]. However, there are few studies concerning in the evaluation of research performances in the field of solid waste [3, 4, 18–21]. To the best of our knowledge, there are no studies that have tried to evaluate research productivity related to solid waste originating from the Arab world.

More recently, scientists from the Arab region have reported an increase in the number of publications in the leading environmental and toxicological journals from Arab researchers [15]. However, the status of solid waste research in this region, until now, has not been reported. Thus, evaluation of Arab research productivity in the field of solid waste may be of interest.

The purposes of this investigation are to analyse research originating from Arab countries in the field of solid waste by using the bibliometric methods, as well as its relative growth rate, collaborative measures, productivity at the institutional level, and the most prolific journals retrieved from the ISI Web of Science database. Bibliometrics is a quantitative analysis aid in the evaluation of research performances in a certain field and allow scientists to identify new lines of research [22–29]. The evaluation of research originating from Arab countries in the field of solid waste provided useful hints about the identification of the main research trends and helped to understand the outlook for progress of this field.

## Methods

### Search strategy

The search for papers to be included in the analysis was carried out using the Science Citation Index (SCI), Web of Science, which is considered one of the most commonly used in such types of studies. We used the bibliometric method as previously described. The keywords used into the Web of Science (WoS) engine to achieve the aim of our study were chosen from previous studies on solid waste [4, 18–21]. “Solid waste*” was used as a search expression to search topic in the SCI over all the previous year’s up to 31 December 2012. This search term included “solid waste” and “solid waste forms” and “solid wastes” and “solid waster.” The topic search in the SCI contains the fields of each paper’s title, abstract, and keywords. In this study, all Arab countries: Yemen; United Arab Emirates (UAE); Tunisia; Somalia; Sudan; Syrian Arab Republic (SAR); Qatar; Oman; Morocco; Mauritania; Libya; Lebanon; Kuwait; Kingdom of Saudi Arabia (KSA); Jordan; Iraq; Egypt; Djibouti; Comoros; Bahrain; and Algeria were used as country keys followed by “solid waste*” key word as a topic. We used the key word “solid waste” because we are concerned with solid waste per se rather than related terminology. Palestine was excluded from our analysis because the WoS database does not categorize it as an independent country yet. The search query appeared like this: (CU = (Yemen) OR CU = (Emirates) OR CU = (Tunisia) OR CU = (Somalia) OR CU = (Sudan) OR CU = (Syrian) OR CU = (Qatar) OR CU = (Oman) OR CU = (Morocco) OR CU = (Mauritania) OR CU = (Libya) OR CU = (Lebanon) OR CU = (Kuwait) OR CU = (Saudi) OR CU = (Jordan) OR CU = (Iraq) OR CU = (Egypt) OR CU = (Djibouti) OR CU = (Comoros) OR CU = (Bahrain) OR CU = (Algeria)) AND TS = “solid waste*” Refined by: [excluding] PUBLICATION YEARS: (2013 OR 2014).

Scientific research productivity after 2012 was excluded from analysis because this period was still open for new journal issues. All data extraction was accomplished on one day (June 7, 2014) to avoid the daily updating on the WoS database. Research performance in the field of solid waste was examined based on a methodology used previously in similar studies [15, 30–35]. The following information was abstracted from the WoS for analysis: (a) total and trends of contributions in solid waste research; (b) research productivity by country; (c) collaboration patterns; (d) journals with their impact factors; (d) research productivity from the most productive institutions; and (e) the citations received by the publications.

### Statistical analysis

The data were recorded by publication year and downloaded into a spreadsheet for descriptive statistics using SPSS software (SPSS version 19.0 for Windows; SPSS, Chicago, IL, USA). Data were expressed as numbers and percentages or as a median (Q1–Q3: interquartile range). Publication activity for each country was adjusted by population size and gross domestic product (GDP), obtained from the online databases of the World Bank [36]. An adjustment index (AI) was calculated using the following formula AI = [raw results from each country / GDP per capita of the country]*1000, where the GDP per capita = GDP/population of the country. This formula has been used in previous bibliometric studies [12, 15, 32].

## Results

**Fig. 1:**
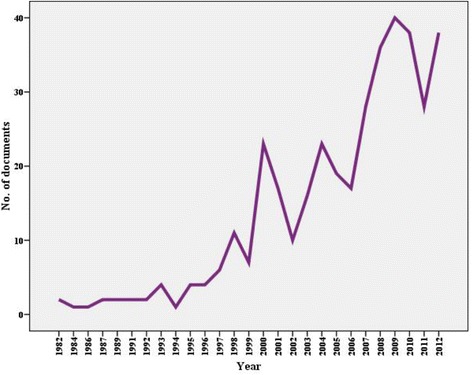
Total articles included in a bibliometric analysis of Arab world publications related to solid waste from 1982 to 2012

The total number of documents related to solid waste obtained by using the key words “solid waste*” in the SCI search engine as topic on the Web of Science without indicating the name of any country and by using the same inclusion criteria was 16,250 documents which represents the overall global research output in the solid waste field. Using the methodology stated above, only 382 (2.35 % of the overall global research output in the solid waste field) documents were retrieved from the Arab countries; comprising 347 (90.8 %) original journal articles, 13 (3.4 %) review articles, and 22 (5.8 %) others, such as editorials or notes. The first article published from the Arab World was in Saudi Arabia, and it was published by Daly and Farooq in the *Journal of the Water Pollution Control Federation* in 1982 [37]. The annual number of documents published in the past three decades (1982–2012) indicated that research productivity demonstrated a noticeable rise during the last decade with peak publications in 2009 (Fig. 1). English language documents were the most common (*n* = 375; 98.2 %), followed by French (*n* = 6), and Spanish language documents (*n* = 1).

The publications share of the top 10 most prolific Arab countries in solid waste research ranges from 2.4 to 22.8 % during 1982–2012. Egypt tops the list, with a publications share of 22.8 % during 1982–2012. Tunisia ranks second (19.6 %), followed by Jordan (13.4 %) (Table 1). After adjusting for economy and population power, Egypt (AI = 27.3) had the highest research productivity. No data related to solid waste were published from Djibouti, Somalia, Comoros, and Mauritania. The total number of citations over the analysed years at the date of data collection was 4,097, with an average of 10.7 citations per document. The median (interquartile range) of citation was 5 (1–13). Iraq achieved the highest median (interquartile range) number of citations with 9 (0–23), followed by 8 (2–14) for Tunisia and 6.5 (2.3–15.3) for Lebanon. The *h*-index of the articles was 31 (31 documents had been cited at least 31 times at the date of data collection). Egypt and Tunisia achieved the highest *h*-index with 15 each, followed by 13 each for Jordan and Morocco. Furthermore, Egypt achieved the highest number of collaborations with international authors with 17 countries, followed by 12 countries for KSA and 10 countries for Jordan (Table 1).

**Table 1:** Bibliometric analysis of the 382 documents from Arab countries associated with solid waste during the period from 1982 to 2012

SCR^a^	Countries	Articles (%)	*h*-index	Median (Q1–Q3) of citation	Average of citation	Collaborations with foreign countries	Number (%)^b^ of documents with international authors	Adjustment index
1^st^	Egypt	87 (22.8)	15	4 (1–12)	10.6	17	27 (31.0)	27.3
2^nd^	Tunisia	75 (19.6)	15	8 (2–14)	12.8	8	31 (41.3)	17.7
3^rd^	Jordan	51 (13.4)	13	5 (1–16)	12.5	10	18 (35.4)	10.3
4^th^	Kuwait	39 (10.2)	8	3 (2–8)	8.7	5	6 (15.4)	0.8
5^th^	Morocco	36 (9.4)	13	6 (2–18)	12	5	24 (66.7)	12.2
6^th^	KSA	25 (6.5)	7	4 (1–9.5)	5.7	12	14 (56.0)	1.0
7^th^	Lebanon	24 (6.3)	8	6.5 (2.3–15.3)	12	2	11 (45.8)	2.5
8^th^	Algeria	20 (5.2)	8	6 (1–12)	9.7	6	11 (55.0)	3.7
9^th^	UAE	10 (2.6)	5	3.5 (1.5–17.5)	9.7	8	5 (50.0)	0.3
10^th^	Iraq	9 (2.4)	5	9 (0–23)	12	3	7 (77.8)	1.4
11^th^	Oman	6 (1.6)	2	1 (0–4.5)	2	3	2 (33.3)	0.3
11^th^	Qatar	6 (1.6)	4	5.5 (3–12.5	8.8	3	4 (66.7)	0.1
13^th^	Syria	4 (1.0)	2	2.5 (0.2–4.8)	2.5	1	1 (25.0)	1.2
14^th^	Bahrain	2 (0.5)	-	-	-	3	2 (100)	0.1
15^th^	Libya	1 (0.3)	-	-	-	1	1 (100)	0.1
15^th^	Sudan	1 (0.3)	-	-	-	0	0 (0.0)	0.6
15^th^	Yemen	1 (0.3)	-	-	-	1	1 (100.0)	0.7
18^th^	Djibouti	-	-	-	-	-	-	-
18^th^	Mauritania	-	-	-	-	-	-	-
18^th^	Somalia	-	-	-	-	-	-	-
18^st^	Comoros	-	-	-	-	-	-	-

*KSA* Kingdom of Saudi Arabia, *SAR* Syrian Arab Republic, *SCR* Standard Competition Ranking, *UAE* United Arab Emirates, *Q1–Q3* lower quartile–upper quartile

^a^Equal countries have the same ranking number, and then a gap is left in the ranking numbers

^b^Percentage of documents with international authors from the total number of documents for each country

In addition, the study recognized 139 (36.4 %) documents from collaborations with 25 non-Arab countries. These collaborations were primarily with authors from France (*n* = 27), followed by the USA (*n* = 26), Italy (*n* = 18), and the UK (*n* = 17) (Table 2). By region, Arab authors mainly collaborated with countries in Europe (22.5 %), especially France, followed by countries in the Americas (9.4 %), especially the USA (Table 2). There was a large variety including 77 subject categories associated to the research topic of solid waste in Journal Citation Reports (JCR) of the ISI. Top 10 subject categories with the most articles are listed in Table 3. Environmental science was the most researched topic, represented by 175 (45.8 %) articles. The second most researched topic was engineering environmental 111 (29.1 %) followed by energy fuels 45 (11.8 %).

**Table 2:** Collaborations between Arab countries and foreign countries in solid waste publications

Collaborating countries^a^	No. of documents (%)
Arab–Europe	86 (22.5)^b^
France	27 (7.1)
Italy	18 (4.7)
England	17 (4.5)
Spain	11 (2.9)
Belgium	8 (2.1)
Germany	3 (0.8)
Austria	1 (0.3)
Denmark	1 (0.3)
Netherlands	1 (0.3)
North Ireland	1 (0.3)
Romania	1 (0.3)
Wales	1 (0.3)
Arab–Americas	36 (9.4)^b^
USA	26 (6.8)
Canada	10 (2.6)
Arab–Other Middle East, Africa	4 (1.1)^b^
Iran	1 (0.3)
Israel	1 (0.3)
Benin	1 (0.3)
Turkey	1 (0.3)
Arab–Southeast Asia	10 (2.6)^b^
Malaysia	10 (2.6)
Arab–Asia-Pacific	10 (2.6)^b^
India	3 (0.8)
Japan	3 (0.8)
Peoples Republic of China	2 (0.5)
South Korea	2 (0.5)
Australia	1 (0.3)
Pakistan	1 (0.3)
Arab–Arab	15 (3.9)

^a^The study identified 139 (36.4 %) documents with 25 countries in Arab–foreign country collaborations

^b^Total exceeds 36.4 % as data are overlapping due to multi-country collaboration

**Table 3:** The top ten ranking of subject categories for published articles associated with solid waste

SCR^a^	Subject categories	*n* (%)^b^
1^st^	Environmental sciences	175 (45.8)
2^nd^	Environmental engineering	111 (29.1)
3^rd^	Energy fuels	45 (11.8)
4^th^	Applied microbiology and biotechnology	44 (11.5)
5^th^	Chemical engineering	35 (9.2)
6^th^	Civil engineering	32 (8.4)
7^th^	Water resources	31 (8.1)
8^th^	Agricultural engineering	24 (6.3)
9^th^	Applied chemistry	14 (3.7)
9^th^	Soil science	14 (3.7)

SCR Standard Competition Ranking

^a^Equal areas of interest have the same ranking number, which leaves a gap in the ranking numbers

^b^Total exceeds 100 % as data are overlapping due to multidiscipline interactions

Articles on solid waste were published in a wide range of 189 peer-reviewed journals. In Table 4, the top 10 most productive journals were summarized with their IF. Twenty seven documents (7.1 %) were published in *Waste Management,* whereas 23 (6.0 %) were published in *Journal of Hazardous Materials*, and 18 (4.7 %) were published in *Bioresource Technology.* As shown in Table 4, all journals were listed in the JCR 2012 and had an official IF.

**Table 4:** The top ten ranking of journals in which articles associated with solid waste were published

SCR^a^	Journal	Frequency (%)	IF^b^
1^st^	Waste Management	27 (7.1)	2.485
2^nd^	Journal of Hazardous Materials	23 (6.0)	3.925
3^rd^	Bioresource Technology	18 (4.7)	4.750
4^th^	Waste Management Research	17 (4.5)	1.047
5^th^	Resources Conservation and Recycling	10 (2.6)	2.319
6^th^	Environmental Technology	9 (2.4)	1.606
7^th^	Journal of Environmental Science and Health Part A. Toxic/ Hazardous Substances and Environmental Engineering	7 (1.8)	1.252
8^th^	World Journal of Microbiology and Biotechnology	6 (1.6)	1.262
9^th^	Desalination	5 (1.3)	3.041
9^th^	Desalination and Water Treatment	5 (1.3)	0.852
9^th^	Kuwait Journal of Science and Engineering	5 (1.3)	0.075

*SCR* Standard Competition Ranking, *NA* not available, *IF* impact factor

^a^Equal journals have the same ranking number, and then a gap is left in the ranking numbers

^b^The impact factor was reported according to the Institute for Scientific Information (ISI) journal citation reports (JCR) 2012

The top 10 most cited articles on solid waste s were listed in Table 5 [38–47]. Table 6 shows a list ranking the top 10 most highly prolific institutions from Arab countries that most frequently published articles related to solid waste. The most productive institution was *American University of Beirut, Lebanon* (6.3 % of total publications), followed by *Kuwait University, Kuwait* (5.5 %), and *Jordan University of Science Technology, Jordan* (5.0 %).

**Table 5:** The top ten ranking of cited articles from the Arab world associated with solid waste in Scopus [38–47]

SCR	Authors-Year	Title	Source title	Cited by
1^st^	Banat et al. 2003 [38]	Evaluation of the use of raw and activated date pits as potential adsorbents for dye containing waters	Process Biochemistry	165
2^nd^	Bouallagui et al. 2005 [39]	Bioreactor performance in anaerobic digestion of fruit and vegetable wastes	Process Biochemistry	124
3^rd^	Hassen et al. 2001 [40]	Microbial characterization during composting of municipal solid waste	Bioresource Technology	101
4^th^	Amin 2009 [41]	Removal of direct blue-106 dye from aqueous solution using new activated carbons developed from pomegranate peel: Adsorption equilibrium and kinetics	Journal of Hazardous Materials	99
5^th^	Amir et al. 2005 [42]	Sequential extraction of heavy metals during composting of sewage sludge	Chemosphere	97
6^th^	Hameed and El-Khaiary 2008 [43]	Batch removal of malachite green from aqueous solutions by adsorption on oil palm trunk fibre: Equilibrium isotherms and kinetic studies	Journal of Hazardous Materials	82
7^th^	Khaled et al. 2009 [44]	Removal of Direct N Blue-106 from artificial textile dye effluent using activated carbon from orange peel: Adsorption isotherm and kinetic studies	Journal of Hazardous Materials	79
8^th^	El-Fadel et al. 2002 [45]	Temporal variation of leachate quality from pre-sorted and baled municipal solid waste with high organic and moisture content	Waste Management	72
9^th^	Bouallagui et al. 2004 [46]	Two-phases anaerobic digestion of fruit and vegetable wastes: bioreactors performance	Biochemical Engineering Journal	60
10^th^	Marafi and Stanislaus 2003 [47]	Options and processes for spent catalyst handling and utilization	Journal of Hazardous Materials	59

*SCR* Standard Competition Ranking

**Table 6:** The top ten ranking of productive institutions from or collaborating with Arab World affiliations during the study period

SCR^a^	Institutions	No. of documents (%)
1^st^	American University of Beirut, Lebanon	24 (6.3)
2^nd^	Kuwait University, Kuwait	21 (5.5)
3^rd^	Jordan University of Science Technology, Jordan	19 (5.0)
4^th^	Alexandria University, Egypt	15 (3.9)
4^th^	Kuwait Institute for Scientific Research, Kuwait	15 (3.9)
4^th^	National Research Centre, Egypt	15 (3.9)
7^th^	University of Tunis El Manar, Tunisia	12 (3.1)
8^th^	Institut National de Recherche Scientifique et Technique, Tunisia	9 (2.4)
8^th^	Université de Toulouse, France	9 (2.4)
10^th^	Centre de Recherches et des Technologies des Eaux Technopole de Borj-Cédria, Tunisia	8 (2.1)
10^th^	Université Chouaib Doukkali, Morocco	8 (2.1)
10^th^	University of Jordan, Jordan	8 (2.1)
10^th^	Université Paul Sabatier Toulouse III, France	8 (2.1)

*SCR* Standard Competition Ranking

^a^Equal institutions have the same ranking number, and then a gap is left in the ranking numbers

## Discussion

The attempts to gather the systematic data to get a panoramic view on this topic were quite few. Bibliometric techniques have been used frequently in many disciplines of sciences to study the scientific research output and trends [15, 23, 25–30, 34]. The SCI database from the Web of Science, the Thomson Reuters, was the most important and frequently used database for the bibliometric research to get a review of scientific accomplishment in many studying fields [23, 34, 48–50]. To our knowledge, this is the first study to analyse the quantity of solid waste-based research by using the total amount of publications and quality of solid waste-based research from Arab world by using the impact factors and *h*-index. Research indicators showed that research activity in this area is insufficient or neglected in some Arab countries. A possible explanation for increasing research activity in the field of solid waste could be that the increase in publication activity reflects a general increase in research and/or publication activity [4, 18–21, 51]. Furthermore, solid waste research productivity has followed the various biomedical research activity in the Arab world in the last decades [12, 14, 15, 31, 34, 51, 52].

The most interesting finding was that the current study demonstrated that Egypt, Tunisia and Jordan, where their total solid waste research output was markedly higher than that in the residual countries. These results seem to be consistent with other research which found that KSA and Egypt had the most prolific Arab countries in biomedical fields [51, 53]. A possible explanation for the dramatic increase in Tunisia publication activity could be due to collaboration with researchers from different foreign countries by investment grant from Washington DC (e.g. sustainable municipal solid waste management project for Tunisia) [54] or from governmental (e.g. Tunisian ministry of higher education scientific research and technology [55].

As expected, both GDP, and population size were the driving force for research activity as noticed in countries like Egypt which is leading the Arab countries in solid waste research. Similar conclusions were reported by other researchers pertaining to research activity in Arab countries [51, 56]. Overall, the annual number for publications from Arab countries in the field of solid waste during the past decades was low. In 2007, the Arab world spent just 0.2 % of its GDP on research and development which is relatively low compared to other neighbouring countries with lower population size such as Israel and Turkey [51, 57–65]. There are several possible reasons for the scarcity of health-related research in most Arab regions were discussed in previous studies [12, 15, 31, 34, 51, 52, 56, 66]. These studies suggested that lack of funding and freedom, and the regional conflict may contribute to shortage of publications related to health in some Arab countries. Also, the lack of industry-academia partnership in applied health research (including government-academia partnerships), and a general weakness in scientific writing may contribute to low scientific research production in most Arab regions [51, 52, 56, 66]. Promotion of research in the field of solid waste in Arab countries needs several strategic goals and serious efforts must be approved by all decision makers and scientists. The strategy should include high-quality training, providing enough funds, improvement research infrastructure, with endorsing excellence [66].

Arab authors collaborated most with countries in Europe region, especially France, Italy and the UK, followed by countries in the Americas, especially the USA. This may be because most Arab academics trained in or graduated from these countries. Additionally, many PhD students from the Arab world got their graduate environmental education in Europe and the Americas, where the concept of environmental sustainability is being emphasized at the research and academic levels. Research collaboration is an imperative method to improve quality and quantity of research [67–69]. A more recent study published in *The Lancet* to improve health-related research in the Arab world recommended that an Arab medical research council – inspired by the US National Institutes of Health, the Medical Research Council in the United Kingdom, and the French Institute of Health and Medical Research (INSERM) – is necessary to establish strategies that promote medical and health research in the Arab world in collaboration with international institutions [66].

In our study, environmental sciences was the most researched topic, represented by around half of the articles, and *Waste Management* was the top active journal. A bibliometric study aimed to evaluate solid waste research at global level, using the literature in the SCI database from 1993 to 2008 found that the most common subject category was environmental science and the top active journal was *Waste Management* [18]. A more recent study using the same method at global level found that research in the field of solid waste focused on engineering and environmental sciences. *Waste Management* published the most articles [4].

In the interpretation of the study results, several limitations should be considered, most of which are similar to the previous studies performed in different bibliometric analyses of the Arab world. One limitation for this approach is that because our search was restricted to SCI-indexed journals, published articles in non-SCI-indexed journals were missed.

## Conclusion

In this study, some significant points have been obtained on the research productivity throughout the period from 1982 to 2012. The number of articles about solid waste increased rapidly in the last 10 years. In total, there are 382 articles in 189 journals listed in 77 SCI subject categories. Environmental sciences was the most researched topic, represented by around half of the articles, and the most productive journal is *Waste Management*. Despite the expected increase in solid waste production from Arab world, research activity about solid waste is still low. Governments must invest more in solid waste research to avoid future unexpected problems. Countries like Egypt must lead Arab researchers in this field given the expertise of Egyptian scientists in this filed. Finally, since solid waste is a multidisciplinary science, research teams in engineering, health, toxicology, environment, geology and others must be formulated to produce research in solid waste from different scientific aspects. The findings from the current study would help researchers from the Arab world to improve the performance and realize more applications in the field of solid waste in the future research.

## Abbreviations

AI: Adjustment index
GDP: Gross domestic product
SPSS: Statistical package for social sciences
ISI: Institute for scientific information
KSA: Kingdom of Saudi Arabia
UAE: United Arab Emirates
SAR: Syrian Arab Republic
USA: United States of America
JCR: Journal citation report
SCR: Standard competition ranking
IFs: impact factors
SCI: Science citation index
